# Special Issue “Innovative Molecular Strategies in Biomedicine”

**DOI:** 10.3390/ijms27198644

**Published:** 2026-09-27

**Authors:** Rui Vitorino

**Affiliations:** Department of Medical Sciences, Institute of Biomedicine—iBiMED, University of Aveiro, 3810-193 Aveiro, Portugal; rvitorino@ua.pt

The paramount transformation in contemporary molecular biomedicine may not be the emergence of a novel therapeutic approach, but rather the evolution of its design unit. Increasingly, engineering focuses not only on a free molecule, material, or device, but on the interface where biological recognition becomes biological activity. This shift is evident in peptide therapies, nanomedicine, regenerative biology, physical medicine, exercise physiology, and advanced in vitro modeling. It provides the conceptual link that unifies the seemingly diverse contributions assembled in this Special Issue, entitled “Innovative Molecular Strategies in Biomedicine”.

This collection addresses five distinct topics: peptide-facilitated heavy-metal detoxification, molecular targeting of damaged central nervous system tissue, keratinocyte molecular responses to magnetic fields and red light, skeletal muscle adaptation in the context of breast cancer and chemotherapy, and a curcumin-derived metal–organic framework for Alzheimer’s disease [1,2,3,4,5]. These investigations originate from several fields independently. Together, they reveal a shared molecular rationale: identify a condition, associate an intervention with that condition, convert the interaction into a pathway-level response, and determine whether the response can be adequately regulated for translation.

This Editorial therefore proposes biointerface programming as a useful hallmark of this Special Issue. The phrase is intentionally used here in a broad yet practical sense. A biointerface is any interface at which an intervention communicates with a biological system: metal ions and peptides, targeting peptides and injury-enhanced extracellular matrix, porous structures and inflammatory pathways, physical stimuli and keratinocytes, exercise levels, and adaptive molecular networks. Programming is not about deterministic control, but about the ability to tune recognition, localization, intensity, timing, and downstream effects. This perspective aligns with the overarching transition from inert biomaterials to adaptive and programmable systems, and from broad interventions to precision approaches tailored to molecular and physiological contexts [6,7,8,9,10,11].

The primary element of this signature is molecular recognition. Peptides provide substantial insight, as their sequence, conformation, charge distribution, and chemical properties can be adjusted to achieve selective interactions, while retaining a size suitable for biological transport and conjugation. These characteristics are increasingly exploited in modern peptide research for targeting, delivery, self-assembly, and direct therapeutic intervention [7,8,12]. This Special Issue extends that reasoning beyond traditional drug development.

Luo et al. examine peptides for heavy-metal remediation and show how amino, carboxyl, thiol, carbonyl, and other functional groups can coordinate with metal ions, form stable complexes, or promote reduction and immobilization [1]. This is not merely an application of peptide chemistry to ecology. It exemplifies a fundamental principle of molecular design, whereby a recognition motif can convert a chemically hazardous condition into a manageable molecular interaction. The same reasoning applies to affinity capture in diagnostics, molecular scavengers, chelating therapies, biosensors, and selective separation methods. In this context, remediation and biomedicine are linked by a common challenge: identifying and removing a molecular entity from a complex milieu.

The issue of translation goes beyond establishing that a peptide can bind a metal ion under defined conditions. The central question is whether binding remains selective and effective in realistic mixtures, whether the peptide is stable, recoverable, and scalable, and whether complex formation reduces biological exposure without introducing new risks. The authors rightly emphasize the need to investigate compound pollution and combinatorial strategies instead of focusing solely on single-metal systems. Such questions imply a growing demand for programmable molecular systems: functionality must withstand complexity.

The second form of recognition is spatial rather than chemical: features of diseased tissue can serve as molecular identifiers. In [3], Castillo et al. present a comprehensive study of the CAQK peptide, investigated as a targeting agent for damaged central nervous system tissue. CAQK interacts with extracellular matrix components that are enriched after injury, including structures associated with chondroitin sulfate proteoglycans. CAQK is typically conjugated to nanoparticles or therapeutic agents. In the 16 studies reviewed, CAQK was used predominantly not as a standalone drug, but as a localization module that enhanced delivery to injured tissue.

This distinction is important. Targeted therapy is often defined by receptors on diseased cells; however, injury, fibrosis, inflammation, and cancer also remodel the extracellular matrix. Thus, the pathological matrix may be viewed as an informational compartment, a molecular representation of tissue status. A targeted ligand that recognizes this altered matrix could turn disease into a manageable interface. This concept is central to the broader evolution of precision nanomedicine, in which the nanoparticle design is increasingly tailored to biological barriers and diverse microenvironments rather than relying on a universal carrier solution [9,13,14,15,16].

Yet the CAQK literature also underscores the difference between evidence of targeting and evidence of therapy. The review highlights variability in doses and study durations, the absence of CAQK-only controls in most experiments, and limited characterization of the exact binding determinants [3]. The indications of adhesion to damaged human cortical tissue are promising but still preliminary. The next stage is not simply adding more payloads to CAQK, but addressing the recognition mechanism, quantifying how conjugation affects biodistribution and clearance, establishing dose–response relationships, and assessing whether localization predicts therapeutic benefit in increasingly human-relevant models. In this setting, specificity must be matched by the specificity of the molecule. The third element of biointerface programming is coupling: building systems in which the carrier and therapy are no longer conceptually distinct. Sun et al. report the use of TAX@medi-MOF-1, a zinc-based metal–organic framework loaded with taxifolin and curcumin, in experimental Alzheimer’s disease research [5]. Curcumin functions as a structural ligand within the framework while retaining pharmacological relevance. The porous structure also accommodates taxifolin. As a result, the framework integrates material synthesis, cargo incorporation, controlled release, antioxidant activity, and modulation of the NLRP3/caspase-1/GSDMD pyroptosis pathway. This design illustrates a broader shift in nanomedicine. Conventional carriers are often intended as physiologically inert transporters. An increasing number of current systems are dynamic structures whose therapeutic mechanisms depend on properties such as chemical composition, porosity, degradation, surface chemistry, or responsiveness. This is particularly appealing in the case of MOFs, whose large surface areas, tunable pore architectures, versatile chemistries, and capacity for multifunctionality create a highly intricate relationship between material design and biological function [17]. At the same time, this complexity heightens the challenge of characterization: particle size, shape, content, cargo positioning, release kinetics, degradation byproducts, protein corona formation, pharmacokinetics, and tissue distribution can all influence biological interpretation [17,18].

Biointerface programming also encompasses interventions without a conventional molecular payload. Woźniacka and colleagues investigated low-frequency magnetic fields, red LED illumination, and their combination in HaCaT keratinocytes [4]. In experimental conditions, magnetostimulation enhanced adhesion and proliferation while altering cytokine release, whereas combined magneto-LED exposure further increased adhesion and proliferation, raised nitric oxide, IL-6, and IL-8 levels, and reduced MMP-9. These findings are noteworthy because keratinocytes are active contributors to inflammatory signaling, re-epithelialization, and extracellular matrix remodeling during wound healing [19,20,21]. The central idea is that physical energy becomes biological only when it is translated into molecular events. In pharmacology, factors such as frequency, intensity, wavelength, duration of exposure, pulse shape, and treatment sequence are analogous to dosage and formulation variables. Biological significance is determined by the condition of the recipient cell and tissue. The contemporary understanding of wound biology emphasizes harmonizing inflammation, proliferation, angiogenesis, matrix deposition, and remodeling rather than optimizing any single process in isolation [19]. Mechanistic investigations in cellular contexts are significant because they connect physical stimulus to the intricate interplay of molecules. The study’s in vitro characteristics are essential for its interpretation. A single layer of keratinocytes cannot reproduce immune cell recruitment, fibroblast activation, vascular responses, matrix dynamics, microbial load, or the diversity observed in chronic wounds. The next phase of investigations should move toward co-cultures, three-dimensional skin equivalents, organotypic systems, and in vivo models, while methodically calibrating physical parameters. This domain remains highly relevant to the initial focus of this Special Issue, particularly with bioprinting and organ-on-chip technology. Advances in 3D bioprinting and organ-on-chip platforms are enabling the creation of organized, human-relevant models that provide simultaneous control over cells, matrix, flow, and architecture [22,23,24,25,26]. Such platforms could enable closer integration between biophysical stimulation and pertinent tissue responses in a therapeutic context.

The publication by Aires et al. expands the concept of molecular intervention. Their analysis examines the loss of skeletal muscle in breast cancer patients undergoing chemotherapy and the potential of exercise to influence the fundamental mechanisms involved [2]. Muscle mass and function are ultimately shaped by the interplay of inflammation, oxidative stress, mitochondrial impairment, proteolysis, autophagy, modified anabolic signaling, and satellite cell dynamics. Physical activity affects numerous processes; however, the kind and extent of adaptation are shaped by the type of exercise, its intensity, duration, frequency, treatment environment, age, menopausal status, existing health issues, and initial functional capacity [27].

Physical activity serves as a quintessential illustration of systems-level programming. The intervention is behavioral, yet the impact is molecular and widespread. This perspective has been further validated by the Molecular Transducers of Physical Activity Consortium (MoTrPAC), which demonstrated extensive time-dependent responses in transcriptomic, proteomic, metabolomic, lipidomic, phosphoproteomic, epigenomic, and immune parameters to endurance training across multiple tissues [28]. Exercise should not be excluded from precision biomedicine. It is a complex exposure whose molecular transducers can be quantified and potentially used to tailor prescriptions [27]. Aires et al. emphasize that most exercise-oncology research has focused on functional outcomes and quality of life, with very little direct molecular examination of skeletal muscle [2]. That disparity is a strategic issue. To move from broad advice to targeted intervention, clinical studies must correlate dosage with molecular response, molecular response with tissue adaptation, and tissue adaptation with outcomes. Such research may reveal response patterns, distinguish aerobic from resistance-specific effects, and determine whether exercise regimens must be customized according to chemotherapy type, inflammatory characteristics, muscle condition, or other quantifiable attributes. The concept mirrors that of a targeted nanoparticle: intervention design yields deeper insights when the biological context is measured rather than assumed. Consequently, the five contributions, despite disciplinary differences, share a common translational grammar. Acknowledgment must be precise enough to function within intricate contexts. Localization should be distinguished from therapeutic causation. Dosage must encompass not only concentration, but also duration of exposure, sequence, physical variables, and the individual patient’s situation. Mechanism must be evaluated at the level at which the intervention claims efficacy. Effectiveness should be coupled with reproducibility, manufacturability, safety, and a reliable trajectory toward human applicability. This framework also clarifies the concept of innovation. A new compound, peptide, or device does not guarantee originality. The truly groundbreaking approach is the one that improves the manageability of a biological process: when it turns a broad interaction into a specific one, when it turns a pathological characteristic into a target, when it integrates structural and pharmacological roles, when it converts a physical signal into a quantifiable molecular reaction, or when it employs molecular phenotyping to tailor a systems intervention. The primary gain in each instance is an increase in resolution, spatial, molecular, temporal, or patient-specific. The broader body of work on precision medicine is evolving in a similar way. Precision goes beyond simply matching a drug to a genotype; it increasingly integrates data on genetics, molecular structures, physiology, behavior, and environmental exposure, while also asking whether the mechanism and benefits of an intervention are tailored to the individual recipient [29,30,31,32]. The biointerface-programming perspective raises a pragmatic design question: which interface is being altered, what data does that interface represent, and what quantifiable outcome indicates successful alteration?

This Special Issue suggests that future advances in molecular biomedicine will be defined less by distinct technological categories and more by the capacity to integrate sensing, targeting, transduction, and adaptation. Peptides may function as recognition components and active agents. The extracellular matrix may become a targeted terrain, therapeutically potent and pharmacologically potent structures. Light and electromagnetic radiation may be treated as standardized molecular inputs. Physical activity may be regarded as a multi-organ molecular perturbation. Bioprinted and organ-on-chip technologies provide human-relevant frameworks for evaluating these interfaces with enhanced physiological relevance [22,23,24,25,26]. The next conceptual advance is adaptive molecular biomedicine, in which interventions are tailored to the biological system’s condition and their efficacy is assessed through quantitative molecular feedback. Multi-omics, high-content imaging, spatial profiling, sophisticated biomaterials characterization, and computational modeling are expected to lead the field, not because of a need to maximize data generation, but because they can reveal the specific interface variables that determine response [29,30,31,32,33,34,35,36]. This is especially important for multifunctional approaches where observable effectiveness may arise from multiple interconnected pathways, and where unintended impacts may be difficult to infer from a single outcome. The enduring implication of “Innovative Molecular Strategies in Biomedicine” is that biomedical technologies are becoming increasingly advanced. Innovation is progressing toward the programmable interface, where chemistry intersects with biology, where materials converge with routes, where energy interacts with cells, and where behavior aligns with molecular networks. Creating an interface with sufficient selectivity, contextual awareness, and translational rigor could become a hallmark of the next era of biomedicine.

## Data Availability

No new data were created or analyzed in this study. Data sharing is not applicable to this article.

## References

[1] Luo Y., Zhang Y., Xiong Z., Chen X., Sha A., Xiao W., Peng L., Zou L., Han J., Li Q. (2024). Peptides Used for Heavy Metal Remediation: A Promising Approach. Int. J. Mol. Sci..

[2] Aires I., Duarte J.A., Vitorino R., Moreira-Gonçalves D., Oliveira P., Ferreira R. (2024). Restoring Skeletal Muscle Health through Exercise in Breast Cancer Patients and after Receiving Chemotherapy. Int. J. Mol. Sci..

[3] Castillo J.A., Le M.N., Ratcliff A., Soufi K., Huang K., Vatoofy S., Ghaffari-Rafi A., Emerson S., Reynolds E., Pivetti C. (2024). Systematic Review of Peptide CAQK: Properties, Applications, and Outcomes. Int. J. Mol. Sci..

[4] Woźniacka R., Lechowska-Liszka A., Stenka B., Bac A., Homa J., Chadzińska M., Ścisłowska-Czarnecka A. (2024). The Effect of Low-Frequency Magnetic Fields with Low Induction and Red LED Light on Keratinocyte Biological Activity-An In Vitro Research Model. Int. J. Mol. Sci..

[5] Sun F., Liu K., Xi E., Zhao Y., Gao N. (2026). Constructing Curcumin-Based Biological Metal-Organic Frameworks (MOFs) for the Treatment of Alzheimer’s Disease Through the Pyroptosis Pathway. Int. J. Mol. Sci..

[6] Song P., Zhou D., Wang F., Li G., Bai L., Su J. (2024). Programmable biomaterials for bone regeneration. Mater. Today Bio.

[7] Warthen J.L., Lueckheide M.J. (2024). Peptides as Targeting Agents and Therapeutics: A Brief Overview. Biomacromolecules.

[8] Guo X.Y., Yi L., Yang J., An H.W., Yang Z.X., Wang H. (2024). Self-assembly of peptide nanomaterials at biointerfaces: Molecular design and biomedical applications. Chem. Commun..

[9] Mitchell M.J., Billingsley M.M., Haley R.M., Wechsler M.E., Peppas N.A., Langer R. (2021). Engineering precision nanoparticles for drug delivery. Nat. Rev. Drug Discov..

[10] Xue L., Thatte A.S., Mai D., Haley R.M., Gong N., Han X., Wang K., Sheppard N.C., June C.H., Mitchell M.J. (2024). Responsive biomaterials: Optimizing control of cancer immunotherapy. Nat. Rev. Mater..

[11] Li J., Mooney D.J. (2016). Designing hydrogels for controlled drug delivery. Nat. Rev. Mater..

[12] Wang L., Wang N., Zhang W., Cheng X., Yan Z., Shao G., Wang X., Wang R., Fu C. (2022). Therapeutic peptides: Current applications and future directions. Signal Transduct. Target. Ther..

[13] Gawne P.J., Ferreira M., Papaluca M., Grimm J., Decuzzi P. (2023). New opportunities and old challenges in the clinical translation of nanotheranostics. Nat. Rev. Mater..

[14] Wilhelm S., Tavares A.J., Dai Q., Ohta S., Audet J., Dvorak H.F., Chan W.C.W. (2016). Analysis of nanoparticle delivery to tumours. Nat. Rev. Mater..

[15] Curvello R., Kast V., Ordóñez-Morán P., Mata A., Loessner D. (2023). Biomaterial-based platforms for tumour tissue engineering. Nat. Rev. Mater..

[16] Debnath K., Las Heras K., Rivera A., Lenzini S., Shin J.-W. (2023). Extracellular vesicle-matrix interactions. Nat. Rev. Mater..

[17] Abánades Lázaro I., Chen X., Ding M., Eskandari A., Fairen-Jimenez D., Giménez-Marqués M., Gref R., Lin W., Luo T., Forgan R.S. (2024). Metal–organic frameworks for biological applications. Nat. Rev. Methods Primers.

[18] Tyagi N., Wijesundara Y., Gassensmith J., Popat A. (2023). Clinical translation of metal–organic frameworks. Nat. Rev. Mater..

[19] Peña O.A., Martin P. (2024). Cellular and molecular mechanisms of skin wound healing. Nat. Rev. Mol. Cell Biol..

[20] Felician M.C.P., Belotto R., Tardivo J.P., Baptista M.S., Martins W.K. (2023). Photobiomodulation: Cellular, molecular, and clinical aspects. J. Photochem. Photobiol..

[21] Mathioudaki E., Rallis M., Politopoulos K., Alexandratou E. (2024). Photobiomodulation and wound healing: Low-level laser therapy at 661 nm in a scratch assay keratinocyte model. Ann. Biomed. Eng..

[22] Leung C.M., de Haan P., Ronaldson-Bouchard K., Kim G.-A., Ko J., Rho H.S., Chen Z., Habibovic P., Jeon N.L., Takayama S. (2022). A guide to the organ-on-a-chip. Nat. Rev. Methods Primers.

[23] Bhatia S.N., Ingber D.E. (2014). Microfluidic organs-on-chips. Nat. Biotechnol..

[24] Ronaldson-Bouchard K., Vunjak-Novakovic G. (2018). Organs on a chip: A fast-track for engineered human tissues in drug development. Cell Stem Cell.

[25] Ingber D.E. (2022). Human organs-on-chips for disease modelling, drug development and personalized medicine. Nat. Rev. Genet..

[26] Huang M.S., Christakopoulos F., Roth J.G., Heilshorn S.C. (2025). Organoid bioprinting: From cells to functional tissues. Nat. Rev. Bioeng..

[27] Noone J., Mucinski J.M., DeLany J.P., Sparks L.M., Goodpaster B.H. (2024). Understanding the variation in exercise responses to guide personalized physical activity prescriptions. Cell Metab..

[28] (2024). MoTrPAC Study Group; Lead Analysts & MoTrPAC Study Group. Temporal dynamics of the multi-omic response to endurance exercise training. Nature.

[29] Hasin Y., Seldin M., Lusis A. (2017). Multi-omics approaches to disease. Genome Biol..

[30] Karczewski K.J., Snyder M.P. (2018). Integrative omics for health and disease. Nat. Rev. Genet..

[31] Aldea M., Friboulet L., Apcher S., Jaulin F., Mosele F., Sourisseau T., Soria J.-C., Nikolaev S., André F. (2023). Precision medicine in the era of multi-omics: Can the data tsunami guide rational treatment decision?. ESMO Open.

[32] Boehm K.M., Khosravi P., Vanguri R., Gao J., Shah S.P. (2022). Harnessing multimodal data integration to advance precision oncology. Nat. Rev. Cancer.

[33] Vandereyken K., Sifrim A., Thienpont B., Voet T. (2023). Methods and applications for single-cell and spatial multi-omics. Nat. Rev. Genet..

[34] Moffitt J.R., Lundberg E., Heyn H. (2022). The emerging landscape of spatial profiling technologies. Nat. Rev. Genet..

[35] Longo S.K., Guo M.G., Ji A.L., Khavari P.A. (2021). Integrating single-cell and spatial transcriptomics to elucidate intercellular tissue dynamics. Nat. Rev. Genet..

[36] Trapnell C. (2024). Revealing gene function with statistical inference at single-cell resolution. Nat. Rev. Genet..

